# Sexual Risk Behavior in HIV-Uninfected Pregnant Women in Western Uganda

**DOI:** 10.1007/s10508-021-02062-0

**Published:** 2021-10-13

**Authors:** Stefanie Theuring, Kenyonyozi Rubagumya, Hannah Schumann, Gundel Harms, John Rubaihayo, Rhoda Wanyenze

**Affiliations:** 1 Institute of Tropical Medicine and International Health, Corporate member of Freie Universität Berlin, Humboldt-Universität Zu Berlin, and Berlin Institute of Health, Charité-Universitätsmedizin, Augustenburger Platz 1, 13353 Berlin, Germany; 2 School of Health Sciences, Mountains of the Moon University, Fort Portal, Uganda; 3 School of Public Health, Makerere University, Kampala, Uganda

**Keywords:** Sexual risk behavior, HIV risk, Uganda, Pregnancy

## Abstract

Our aim was to identify sexual risk behavior among HIV-negative pregnant women in Kabarole District, Uganda, by conducting a cross-sectional study among 1610 women within three healthcare settings. One in six women engaged in HIV-specific risk behaviors including multiple sexual partners or alcohol abuse; 80% of the pregnant women reported to generally abstain from using condoms. In multivariate analysis, predictors of sexual risk behavior included being a client of the public health facilities as compared to the private facility (AOR 3.6 and 4.8, *p* < 0.001), being single, widowed or divorced or not cohabiting with the partner (AOR 4.7 and 2.3, *p* < 0.001), as well as higher household wealth (AOR 1.8, *p* < 0.001) and lack of partner status knowledge (AOR 1.6, *p* = 0.008). Self-estimated risk perception was linked with engagement in HIV-related risk behaviors except for alcohol abuse. Our findings indicate that reducing risky behaviors in pregnancy in order to prevent HIV should be a high-priority public health concern.

## Introduction

While the majority of new HIV infections in Sub-Saharan Africa generally occur in women (UNAIDS, 2020), especially pregnant women may have an elevated risk for HIV incidence compared to the general population (Drake et al., 2014; Kinuthia et al., 2010; Moodley et al., 2009). A study in Rakai, Uganda, revealed 2.3 seroconversions per 100 persons-years in pregnant women compared to 1.1 per 100 person-years in non-pregnant women (Gray et al., 2005). A recent study in Kabarole District, Uganda, showed HIV incidence rates up to 3.0 per 100 women-years in urban and 5.2 per 100 women-years in rural pregnant populations (Schumann et al., 2020). These numbers are particularly worrisome when considering that seroconversion in pregnancy inherits a high risk for vertical transmission, due to a viral load peak during acute infection (Johnson et al., 2012). Assumedly, 43% of all mother-to-child transmissions are a result of seroconversion during pregnancy or breastfeeding (Johnson et al., 2012). Thus, pregnant women do not only represent a high-risk group for new HIV infections, but also are high-risk vertical transmitters in case of seroconversion. 

It is not entirely clear why pregnant women are at increased risk for HIV infection. On the one hand, pregnancy itself may increase the biological susceptibility of a woman for HIV infection through immunological factors or hormonal changes altering genital mucosal surface. On the other hand, pregnant women may have an elevated risk due to specific sociodemographic, sexual and partnership characteristics, potentially influencing their risk for HIV acquisition (Kinuthia et al., 2010). Yet, there is rather limited literature available on sexual risk behavior in pregnancy. A study in South Africa pointed out that 60% of interviewed pregnant women did not know the HIV status of their partner and an equal rate had not used condoms during the past months; 20% of the woman in this study reported multiple sex partners in the past year (Peltzer & Mlambo, 2013). Several other studies reported accordingly that condom use was less frequent in pregnant women. (Gray et al., 2005; Jones et al., 2011; Keating et al., 2012). This might be a result of omitting condoms as a purely contraceptive method without considering prevention of sexually transmitted diseases including HIV (Peltzer & Mlambo, 2013), possibly also linked with a weaker condom-negotiating position of the woman toward the partner or husband based on this rationale. Overall, while mechanisms for HIV-positive pregnant women to prevent vertical transmission have received a lot of attention in the past decades, primary prevention among those antenatal care (ANC) clients tested HIV-negative has largely remained out of focus. Offering repeat testing during pregnancy, as it is recommended in many countries including Uganda (Uganda MoH, 2016), will help identify some seroconverted women, but is not primarily contributing to HIV prevention in pregnancy.

In order to inform and guide effective prevention interventions, it is crucial that we gain better understanding of behavioral specificities in pregnancy potentially influencing the risk for HIV seroconversion. Hence, the aim of this study was to shed light on sexual risk behavior of HIV-negative women during pregnancy and factors associated with this behavior, in order to be able to tailor HIV prevention programs fitting specific needs of this vulnerable population group. Our study was located in Kabarole District, Western Uganda, an area highly affected by the HIV epidemic (Rubaihayo et al., 2010; Uganda AIDS Commission, 2016). HIV-positive pregnant women in Kabarole District receive care and treatment following a well-established health service algorithm including Option B+ , i.e., lifelong antiretroviral therapy (Schnack et al., 2016), but similar to the larger global picture, in this setting, ANC clients also largely drop out of the focus of HIV-related attention as soon as tested HIV-negative.

## Method

Within the larger frame of a cross-sectional study on HIV incidence among pregnant women in Western Uganda (Schumann et al., 2020), we performed a retrospective assessment of sexual risk behavior among HIV-negative women during their current pregnancy. Primary outcome parameter was the prevalence of risk behavior in pregnant women. Secondary outcome parameters were sociodemographic and economic predictors for sexual risk behavior and the linkage between women´s own risk perception and risk behavior.

### Study Setting

The study was located in the rural setting of Kabarole District, which has an estimated population of 450,000. Despite considerable efforts, HIV remains a major problem in this district, with Fort Portal Municipality belonging to one of the most HIV-affected areas in Uganda (Rubaihayo et al., 2010). In previous studies, HIV prevalence in pregnant women in Fort Portal, the district capital, was found to be about 10% in 2013 (Schnack et al., 2016). Three hospitals in Fort Portal serve the population in Kabarole District, in addition to approximately 40 further health centers level I–IV (Uganda Bureau of Statistics, 2016).

Our research sites included the Regional Referral Hospital Fort Portal, also known as Buhinga, the private catholic Holy Family Virika Hospital in Fort Portal, and Kibiito Health Center IV in a rural area 30 km south of Fort Portal. The three hospitals were selected purposively using a maximum variation approach (Palinkas et al., 2015) and are covering differences in scope (governmental/private, level of care) and facility size. Virika and Kibiito each attend to around 100, Buhinga to around 250 new ANC clients per month. All three sites offer standard ANC services, obstetric care, postpartum care and HIV care free of charge. ANC clients are tested for HIV at first ANC encounter and enrolled in PMTCT services if HIV-positive. If HIV-negative, they are re-tested after three months according to the national and WHO guidelines (WHO, 2010). None of the three facilities offered specific HIV education or other prevention interventions for HIV-negative pregnant women at the time of the study.

### Participants and Procedure

Recruitment of study participants took place in ANC and maternity wards of the three facilities. Through this, we aimed at enrolling as many women as possible within the recruitment period, including those only attending ANC, but not delivering in hospital, as well as those not attending more than one ANC visit or attending ANC elsewhere, but delivering at the study sites. This recruitment strategy was designed to help avoiding selection bias associated with facility-based deliveries or with ANC late-presenters. Data collection took place between June and November 2017. At each of the three study sites, there was one nurse in charge of the study who had been trained in study procedures and who instructed other staff.

General eligibility criteria included existing pregnancy with being in the third gestational trimester or early post-delivery stage while still being hospitalized after giving birth, and having tested HIV-negative at the first ANC visit in this pregnancy. Including women at ≥ 28 gestational weeks ensured to cover a sufficiently large period of pregnancy when asking about sexual risk behavior. Other eligibility criteria were age of 15 years (emancipated minors; Uganda National Council for Science and Technology, 2014) or above and written informed consent to participate in the study. Women were recruited into the study subsequently to their routine health care (ANC or delivery-related).

### Measures

After obtaining informed consent, a study nurse conducted an interview based on a structured questionnaire on sociodemographic factors, like age, education and marital status, as well as behavioral information, such as number of sexual partners, use of condoms, or alcohol abuse. In addition, women were asked to self-assess their perceived risk for acquiring HIV infection in four categories from “high risk” to “not at risk.” For evaluating their socioeconomic status (SES), participants reported the availability of certain items within the household, including radio, fridge, a motorbike or car, electricity, tap water, a cupboard, TV, cattle and a mosquito net, resulting in a wealth score ranging from 0–9.

From the behavioral variables reported in the interviews, we built a dichotomous category for presence or absence of risk behavior in pregnancy, with any one of the following variables leading to inclusion in the “risk behavior” group: two or more sexual partners; at least one incidence of unprotected sex with an unknown person; alcohol abuse (i.e., habit of repeated excessive alcohol drinking); at least one incidence of sex under the influence of drugs or alcohol. These variables were chosen because they were independent predictors of HIV seroconversion in pregnancy in preceding data analysis within the same cohort (Schumann et al., 2020), they also comply with variables defining the “at-risk” group in other research (Palomino González et al., 2019). The study nurses asked for engagement in the respective behaviors since the start of 2017, hence, covering six months at the onset of data collection in June. Additionally, study nurses probed women specifically as to engagement in those behaviors during pregnancy. In addition to the dichotomous risk behavior category, we also built a risk behavior score ranging from 0–4 according to the number of risk behaviors a person reported.

### Data Analysis

A descriptive analysis was performed, calculating frequencies and percentages for the total cohort as well as for the three health facilities. In bivariate analysis, we identified significant associations of sociodemographic characteristics with engagement in risk behavior using Pearson´s chi-square test. *p*-levels below 0.05 were considered as statistically significant. To adjust for potential confounders, we performed multivariate analysis on those factors showing significance in bivariate analysis. For this purpose, we used a binary logistic regression model based on the dichotomous risk behavior category and compared this with results of an ordered logistic regression model based on the risk behavior score. For both models, we report adjusted odds ratios and 95% confidence intervals. To test the association between own risk perception regarding HIV acquisition and the risk behavior score, we used a chi-square-contingency table. To assess the link of sexual risk behaviors and perceived risk to acquire HIV, we calculated adjusted odds ratios using ordered logistic regression.

## Results

From the recruited 1610 clients, 703 (43.7%) were from the public Buhinga Hospital, 408 (25.3%) from the rural Kibiito Health Center, and 499 (31.0%) from the private Virika Hospital. Altogether, 884 (55%) women were recruited in ANC and 726 in the maternity ward. Sociodemographic as well as risk-behavioral parameters in the three different facilities are indicated in Table 1. The majority (55.7%) of participants were younger than 25 years. Only 14% did not have a partner, but about half of the woman in marriage or a relationship were not permanently cohabiting with their partner. Almost two-thirds estimated their own risk for HIV acquisition as very low or nonexistent. About 18% of all participants stated not to know their partner´s HIV status. About 72% stated to be sexually active at this point. Almost 80% of all clients reported that they would generally never use condoms.

**Table 1:** The sociodemographic characteristics, risk perception, and risk behavior of the study participants

Variable	Total	Buhinga (urban public)	Kibiito (rural public)	Virika (urban private)
	*n* (%)			
Total study population (*n* = 1610)	1610 (100)	703 (43.66)	408 (25.34)	499 (30.99)
Age groups (*n* = 1604)				
15–24 years of age	893 (55.7)	427 (60.9)	240 (59.3)	226 (45.4)
25–34 years of age	580 (36.2)	226 (32.3)	131 (32.4)	223 (44.8)
35–50 years of age	131 (8.17)	48 (6.9)	34 (8.4)	49 (9.8)
Marital status (*n* = 1608)				
Married or cohabiting	625 (38.9)	222 (31.6)	229 (56.1)	174 (34.9)
Non-cohabiting couple	754 (46.9)	346 (49.3)	132 (32.4)	276 (55.4)
Single, widowed or divorced	229 (14.2)	134 (19.1)	47 (11.5)	48 (9.6)
Education (*n* = 1606)				
Primary or less	848 (52.8)	391 (55.7)	281 (69.0)	176 (35.4)
Secondary or more	758 (47.2)	311 (44.3)	126 (31.0)	321 (64.6)
Wealth score (*n* = 1610)				
0–3	574 (35.7)	218 (31.0)	240 (58.8)	116 (23.3)
4–9	1036 (64.4)	485 (69.0)	168 (41.2)	383 (76.8)
Partner age (n = 1597)				
14–24 years of age	341 (21.4)	163 (23.5)	103 (25.3)	75 (15.2)
25–34 years of age	794 (49.7)	348 (50.1)	201 (49.4)	245 (49.5)
35–60 years of age	462 (28.9)	184 (26.5)	103 (25.3)	175 (35.4)
Partner education (*n* = 1590)				
Primary or less	643 (40.4)	268 (38.8)	242 (59.6)	133 (26.9)
Secondary or more	947 (59.6)	422 (61.2)	164 (40.4)	361 (73.1)
Own HIV risk perception (*n* = 1608)				
High	131 (8.2)	53 (7.5)	49 (12.0)	29 (5.8)
Some	419 (26.1)	205 (29.2)	111 (27.3)	103 (20.7)
Very low	396 (24.6)	242 (34.4)	38 (9.3)	116 (23.3)
Not at risk	662 (41.2)	203 (28.9)	209 (51.4)	250 (50.2)
No. of sexual partners in the ongoing year (*n* = 1578)				
One or less	1337 (84.7)	528 (76.4)	352 (88.2)	457 (93.7)
Two or more	241 (15.3)	163 (23.6)	47 (11.8)	31 (6.4)
Unprotected sex with unknown person (*n* = 1610)				
Yes	46 (2.9)	26 (3.7)	16 (3.9)	4 (0.8)
No	1564 (97.1)	677 (96.3)	392 (96.1)	495 (99.2)
Alcohol abuse (*n* = 1610)				
Yes	92 (5.7)	69 (9.8)	19 (4.7)	4 (0.8)
No	1518 (94.3)	634 (90.2)	389 (95.3)	495 (99.2)
Sex under influence of drugs or alcohol (*n* = 1610)				
Yes	17 (1.1)	7 (1.0)	10 (2.5)	0
No	1593 (98.9)	696 (99.0)	398 (97.6)	499 (100.0)
HIV Status of partner known (*n* = 1607)				
Yes	1318 (82.0)	576 (82.1)	304 (74.7)	438 (88.0)
No	289 (18.0)	126 (18.0)	103 (25.3)	60 (12.1)
Currently sexually active (*n* = 1604)				
Yes	1154 (72.0)	534 (76.0)	243 (59.7)	377 (76.3)
No	450 (28.1)	169 (24.0)	164 (40.3)	117 (23.7)
Condom use when sexually active (*n* = 1571)				
Always	22 (1.4)	7 (1.0)	8 (2.0)	7 (1.4)
Sometimes	144 (9.2)	61 (8.9)	49 (12.2)	34 (7.0)
Rarely	150 (9.6)	84 (12.3)	18 (4.5)	48 (9.9)
Never	1255 (79.9)	532 (77.8)	327 (81.3)	396 (81.7)

Among our risk-behavior categories, 15.3% of all women stated they had two or more sexual partners in the current year, 5.7% reported alcohol abuse, 2.9% said they had at least one incidence of unprotected sex with an unknown person, and 1% stated that they had had sex under the influence of drugs or alcohol. From all 1610 participants, 269 (16.7%) reported to engage in at least one behavior category implicating risk of HIV acquisition; one-third of these (*n* = 89) were involved in two or more risky behavior categories.

### Association of Sociodemographic Characteristics with Reported Risk Behavior

Bivariate analysis (Table 2) showed significant differences between women who engaged in HIV risk behavior and women who did not, related to their age group, marital status, wealth category, and knowledge on partner status. While among clients of the large urban public Buhinga Hospital, 25.0% reported to engage in risk behavior, this applied to 14.1% in the rural public Kibiito HC, and only to 6.6% in the private, catholic Virika Hospital.

**Table 2:** Association of sociodemographic characteristics with reported risk behavior

Variable	Participants reporting risk behavior^a^	Participants reporting no risk behavior	*p *value ^b^
Total study population (*n* = 1610)	269 (16.7%)	1341 (83.3%)	–
Age group (*n* = 1604)			
15–24 years of age	173 (19.4%)	720 (80.6%)	
25–34 years of age	84 (14.5%)	496 (85.5%)	0.003
35–50 years of age	12 (9.2%)	119 (90.8%)	
Facility (*n* = 1610)			
Buhinga (urban public)	176 (25.0%)	527 (75.0%)	
Kibiito (rural public)	60 (14.1%)	348 (85.9%)	< 0.001
Virika (urban private)	33 (6.6%)	466 (93.4%)	
Marital status (*n* = 1608)			
Married or cohabiting	58 (9.3%)	567 (90.7%)	
Non-cohabiting couple	133 (17.6%)	621 (82.4%)	< 0.001
Single, widowed or divorced	78 (34.1%)	151 (65.9%)	
Education (*n* = 1606)			
Primary or less	141 (16.6%)	707 (83.4%)	0.890
Secondary or more	128 (16.9%)	630 (83.1%)	
Wealth score (*n* = 1610)			
0–3	77 (13.4%)	497 (86.6%)	0.008
4–9	192 (18.5%)	844 (81.5%)	
Partner HIV status (*n* = 1607)			
Known	196 (14.9%)	1122 (85.1%)	< 0.001
Unknown	73 (25.3%)	216 (74.7%)	
Partner age (*n* = 1597)			
14–24 years of age	62 (18.2%)	279 (81.8%)	
25–34 years of age	140 (17.6%)	654 (82.4%)	0.100
35–60 years of age	62 (13.0%)	400 (87.0%)	
Partner education (*n* = 1590)			
Primary or less	99 (15.4%)	544 (84.6%)	0.396
Secondary or more	161 (17.0%)	786 (83.0%)	

^a^ Risk behavior is defined as mentioning of at least one of the following behaviors in the ongoing year, i.e., during pregnancy: number of sexual partners > 1, unprotected sex with unknown persons, alcohol abuse and sex under the influence of drugs or alcohol

^b^ Pearson´s chi-square test

In accordance, both logistic regression models (Table 3) identified the following factors to be independent predictors of engagement in HIV risk behavior: being a client at Buhinga or Kibiito; living in a non-cohabiting relationship or being single, widowed or divorced; reporting a higher wealth score; and reporting unknown partner status. Age was no longer a statistically significant influencing factor of risk behavior in multivariate analysis. ANC clients attending Buhinga and Kibiito were about 4 times more likely to report risk behavior compared to clients attending Virika Hospital. Women being single, widowed or divorced were at almost fivefold risk of reporting risk behavior engagement, while women who were in a partnership, but not permanently cohabiting with their partner still were twice as likely to engage in risk behavior compared to women living with their partner.

**Table 3:** Association of sociodemographic characteristics with sexual risk behavior during pregnancy: Logistic regression models^a^

Variable	Model 1: Logistic regression Risk behavior as binary variable (*n* = 1569^b^)	Model 2: Ordered logistic regression Risk behavior as ordinal variable (*n* = 1569^b^)
	AOR (95% CI), *p *value	
Facility^d^		
Virika (reference)	–	–
Kibiito	3.600 (2.212–5.860), < 0.001	3.794 (2.331–6.173), < 0.001
Buhinga	4.747 (3.157–7.138), < 0.001	4.921 (3.272–7.400), < 0.001
Age^c d^	1.005 (0.980–1.032), 0.690	1.011 (0.985–1.037), 0.404
Marital status^d^		
Married or cohabiting (reference)	–	–
Non-cohabiting couple	2.304 (1.629–3.258), < 0.001	2.441 (1.727–3.451), < 0.001
Single, widowed or divorced	4.654 (3.006–7.207), < 0.001	5.042 (3.269–7.779), < 0.001
Education^d^		
Primary or less (reference)	–	–
Secondary or more	1.023 (0.756–1.383), 0.884	1.007 (0.748–1.357), 0.961
Wealth score^c d^	1.188 (1.096–1.289), < 0.001	1.196 (1.103–1.296), < 0.001
Partner HIV status^d^		
Known (reference)	–	–
Unknown	1.594 (1.129–2.252), 0.008	1.655 (1.180–2.321), 0.004
Overall model pseudo-R-squared value, *p*-value	0.1115, < 0.001	0.0888, < 0.001
Pearson goodness-of-fit test: Estimate, *p*-value	1214.86, 0.0559 ^e^	–
Hosmer–Lemeshow test: Estimate, *p*-value	–	31.341, 0.6455^e^

^a^Four risk factors for HIV incidence in pregnancy (number of sexual partners > 1, unprotected sex with unknown persons, alcohol abuse, and sex under influence of drugs or alcohol) were evaluated using two scoring systems, a binary and an ordinal one. The binary score included two values, where 0 = participant reporting no risk behavior and 1 = participant reporting one risk behavior or more; the ordinal score included five ordinal values, where 0 = participant reporting no risk behavior and 4 = participant reporting all four risk behaviors

^b^Observations with missing values were excluded in the two regression models

^c^Continuous variable

^d^The two models are showing matching results

^e^*p* value is showing the model to be a good fit

OR: odds ratio

CI: confidence interval

### Own Risk Perception of HIV Acquisition and Risk Behavior

**Fig. 1:**
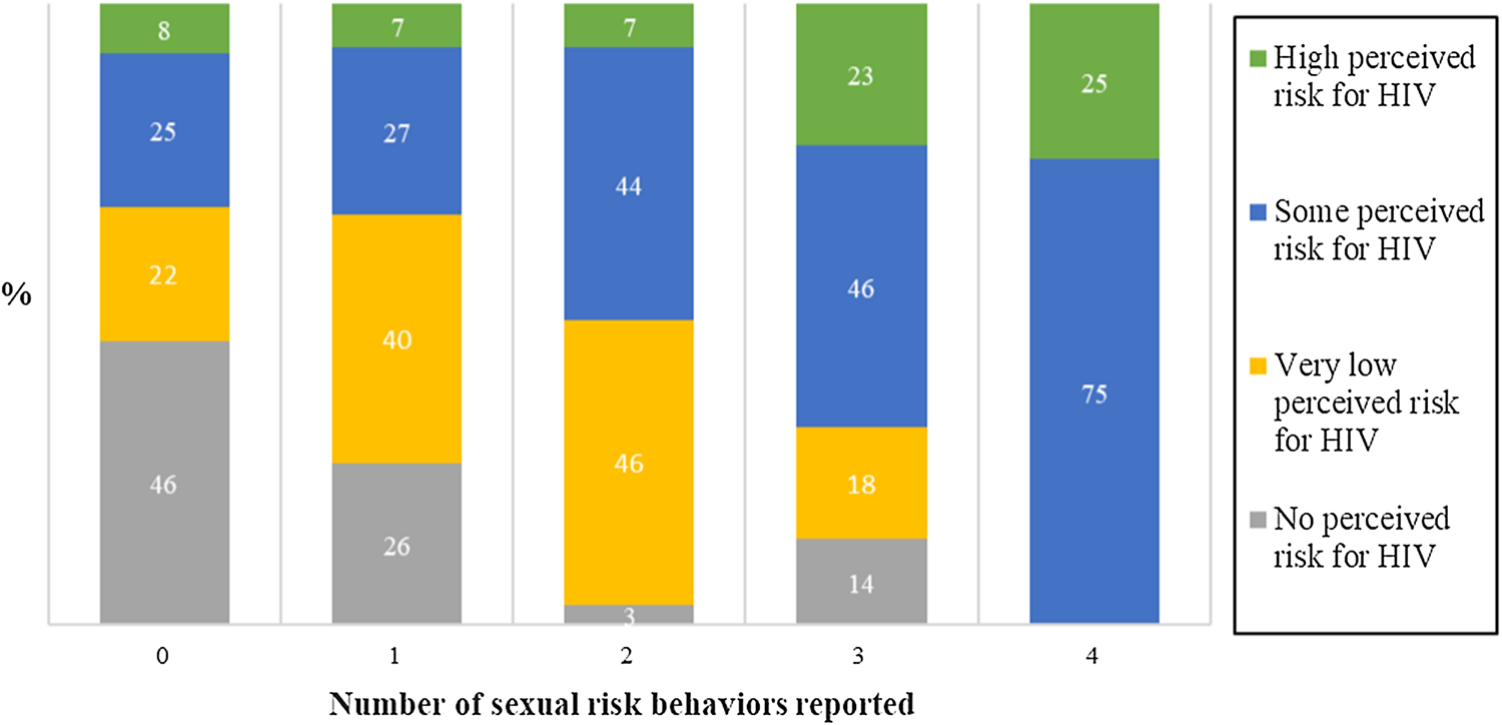
Sexual risk behavior score and own perceived risk of acquiring HIV. Pearson´s chi-square *p* < 0.001

The level of study participants´ self-perception of HIV risk was significantly linked to the number of risk behaviors a person engaged in (Fig. 1). When analyzing independent influence of four different HIV risk behavior variables on the level of own risk perception, we found that sex under the influence of drugs or alcohol, unprotected sex with unknown persons, and two or more sexual partners in the ongoing year were independently associated with an increased risk perception on a statistically significant level. Alcohol abuse was the only factor not significantly associated with risk perception (see Table 4 and Fig. 1).

**Table 4:** Association of participants’ own HIV risk perception with their sexual risk behaviors, ordered logistic regression

Risk Behavior	Own risk perception, ordered scale AOR (95% CI), *p* value
Alcohol abuse	1.474 (0.969–2.241), 0.070
Unprotected sex with unknown person	2.232 (1.192–4.180), 0.012
Sex under influence of drugs or alcohol	2.882 (1.126–7.376), 0.027
Number of sexual partners > 1	1.440 (1.104–1.880), 0.007
Overall model pseudo-R-squared value, *p *value	0.0139, < 0.001

## Discussion

Within a large sample of HIV-negative pregnant women, our research assessed HIV-related risk behavior and identified groups of women particularly prone to high-risk behavior. While almost one in five women stated that her partner´s HIV status was unknown to her, the vast majority of our sample also reported to generally not use condoms. Overall, one in six pregnant women reported engagement in at least one risk-linked behavior in pregnancy.

As anticipated prior to selecting the study sites, the three different health settings showed differences in the clientele characteristics and in behaviors. Clients at the public, governmental facilities were about four times as likely to engage in risk behavior as compared to the private hospital. A Kenyan study on urban and rural differences in sexual risk behavior (Dodoo et al., 2007) found that within the subgroup of poorer clients, urban poor population was significantly more prone to risky sexual behaviors than rural poor population. Assuming that public facility clients are usually poorer than private facility clients, as also mirrored in the wealth score distribution in our cohort, this precisely reflects findings in our three facilities: “urban poor” clientele in Buhinga was much more engaged in risk behavior (25%) than “rural poor” clientele in Kibiito (14%), while least risk behavior was displayed in the private facility Virika (7%). This is a highly relevant meso-level finding for prevention service implementers, implying that especially in public facilities and in rural areas, where pregnant women also tend to be younger and less educated, the focus on risk behavior information and prevention measures should be strongly sharpened. In terms of cost-effectiveness, systematically targeting entire healthcare settings which are known for vulnerable clientele could have implementation advantages over identification of individual at-risk clients at such facilities.

The fact that women who did not know their partner´s status were showing more risk behavior could be a result of an underlying factor of general carelessness or lack of health education in this group. Women´s marital and cohabiting status was also a significant predictor of risk behavior engagement. Women in a partnership, but not permanently living with their partner, as well as the single, divorced, or widowed were at double, respectively, fivefold increased risk for potentially harmful sexual behavior. Widows and divorced women have been described as particularly vulnerable for HIV in previous research in several African countries (Abimanyi-Ochom, 2011; Tenkorang, 2014), which can partly be explained by financial hardship and sexually exploitative societal customs this group of population is confronted with, for instance, in the case of widow inheritance by other family members. A conscientious health policy response needs to protect women from exposure to such detrimental conventions. Our findings also support research results from Thailand, where among a sample of factory workers, risk behavior was fivefold higher in those not cohabiting with a partner (Hong & Thepthien, 2017), probably because of the lack of social control when the partner is away from home. The Thai study also found an effect from alcohol abuse, increasing risk behavior especially among those not cohabiting with a partner.

On a micro-level, as opposed to the aforementioned meso-level of healthcare settings, a higher wealth score in individual persons turned out to be a predictor of HIV risk behavior. For a long time, it was contrarily argued that poverty is the main underlying factor of HIV risk (Fenton, 2004). This was undermined by several studies stating that, somewhat counterintuitively, household wealth can in fact be strongly associated with HIV prevalence (Abimanyi-Ochom, 2011; Parkhurst, 2010; Shelton et al., 2005) possibly by simply enabling someone to have more sexual partners. While those studies looked at HIV prevalence and could not exclude a bias of wealthier people with HIV potentially living longer than less wealthy people, hence showing higher prevalence rates, the findings of our study support the recognition that wealthier individuals within all different types of institutional settings actually are more likely to engage in risk behavior. Shelton et al. (2005) argued that wealth is an important prerequisite for social networks, mobility and time, all of which are key enablers for having concurrent partnerships, and they point out that this connection was even stronger in women than in men in data from East Africa. We argue that higher wealth might also enable individuals to excessively consume alcohol; however, these two factors were not associated on a significant level in our study.

Despite the fact that general discouragement of alcohol consumption in pregnancy is taking place in antenatal counseling because of the teratogenic effect on the fetus, in our sample, alcohol abuse was present to some extent, reaching 10% in the urban public health facility. Those rates may still be an underestimate caused by social desirability bias. Other studies have equally observed high acceptability of alcohol consumption during pregnancy in Sub-Saharan African countries (Mpelo et al., 2018; Watt et al., 2014). Watt et al. described alcohol as a coping strategy with stressors during pregnancy, but also as a way to maintain social connections during this time. Interestingly, in the context of HIV risk awareness, reported alcohol abuse was the only risk factor that was not leading to a higher self-awareness of individual risk for HIV in our study participants. While there might be a general societal problem of sufficient consciousness of risks emerging from alcohol consumption in pregnancy (Corrales-Gutierrez et al., 2019), this finding implies that there might be a particular lack of knowledge on the potentially harmful outcomes of impaired sexual decision-making after alcohol consumption. Health service implementers should direct more attention to the problem of alcohol drinking during pregnancy, creating alertness not only regarding detrimental effects on the unborn, but also regarding the associated risk for a woman to get infected with HIV. Since alcohol consumption is a behavior deeply rooted within societies and cultures, approaches to target this matter should not focus on health facilities alone, but also on other public sceneries like bars and alcohol-serving settings, which might offer important opportunities to identify and counsel women at risk of drinking during pregnancy (Watt et al., 2014). Overall, the HIV risk self-perception in our cohort was significantly related to engagement in sexual risk behaviors, which confirms previous research from Uganda (Kibombo et al., 2007; Palomino Gonzáles et al., 2019) showing that knowledge on HIV risk behaviors and awareness of the individual risk it might create is present to some extent.

The main strength of this study is that it explores sexual risk behavior among a large cohort of HIV-negative, pregnant women in a high-prevalence setting in Uganda. As a limitation, we cannot exclude some underreporting of risk behaviors due to social desirability bias, with pregnant women possibly feeling obliged to deny detrimental behavior in front of ANC staff. We tried to mitigate this bias by framing the reporting period as “since the beginning of this year,” rather than “during this pregnancy,” as pregnant women might have felt enabled to make more open statements through this phrasing. Staff were then probing women´s answers with regard to conduct during pregnancy, to avoid capturing pre-pregnancy behavior. Another limitation is that women in our study might have overreported knowledge of partner status and underreported positive HIV partner status to avoid social stigmatization. Positive partner status might be a significant risk factor for HIV and could influence risk behavior; however, due to suspected underreporting, we did not consider this factor for analysis.

### Conclusion

Our study fills a gap of knowledge on sexual risk behavior during pregnancy by analyzing this behavior within a large cohort of HIV-negative women in Kabarole District, Uganda. One in six women engaged in HIV-specific risk behaviors, and the majority of the women reported to generally abstain from using condoms. Public hospitals, especially in an urban environment, featured clientele with highest rates of risk behaviors. Women´s marital and cohabiting status was an important associated factor of risk behavior engagement. Self-estimated individual risk to obtain HIV concurred with engagement in most HIV-specific risk behaviors, but there seems to be a lack of consciousness regarding the effect abusive alcohol consumption can have on sexual health outcomes.

Our findings indicate that HIV prevention in pregnancy is a highly relevant public health concern, and health facilities in settings with high HIV incidence rates should target improved prevention activities at large scale. Individual risk counseling and support should be provided for specific risk groups including women who are widowed, divorced or do not cohabit with their partner. In addition, campaigns to increase awareness on alcohol abuse and the potential danger arising from it, for the fetus but also with respect to HIV infection, are urgently needed.
